# Evaluation of Nurses’ attitudes, behaviors, and barriers toward pressure ulcer prevention in neonatal and pediatric intensive care units

**DOI:** 10.3389/fped.2024.1455950

**Published:** 2024-11-06

**Authors:** Osama Elshahat Mostafa, Nazik M. A. Zakari, Marwa Al Salem

**Affiliations:** Department of Nursing, College of Applied Sciences, AlMaarefa University, Riyadh, Saudi Arabia

**Keywords:** pressure ulcer prevention, nurse attitudes, barriers to care, neonatal intensive care, pediatric intensive care

## Abstract

**Introduction:**

Pressure ulcers are a significant concern in pediatric intensive care units, with prevalence rates ranging from 0.8% to 27%. They pose serious physical and psychological challenges, particularly in neonatal and pediatric intensive care units (NICU and PICU). This study explores nursing strategies, attitudes, and barriers toward pressure ulcer prevention in NICU and PICU settings.

**Methods:**

Using a descriptive study design, data were collected from 80 nurses working in NICU and PICU through validated questionnaires, including a demographic profile and a pressure ulcer survey. Descriptive statistics were employed to calculate mean scores and percentages, while inferential statistics assessed associations between variables.

**Results:**

The study revealed specific nursing strategies, along with significant barriers and attitudes toward pressure ulcer prevention in NICU and PICU. The total attitude mean score was 3.57, with the highest positive response (mean = 4.29) for “most pressure sores can be avoided”, and the highest negative response (mean = 3.86) for “pressure sore prevention is a low priority for me”. Among participants, 72.5% conducted risk assessments on all patients, 60% had written prevention care plans, and 76.3% implemented preventive strategies. Barriers such as staff shortages and time constraints were reported by 76.2% of nurses. Multivariate analysis indicated that nurses with more than 10 years of qualification (OR = 3.67) and permanent staff with over 10 years of employment (OR = 4.31) were significantly more likely to engage in preventive practices. The use of a pressure ulcer grading tool (OR = 2.49, *P* < 0.05) and participation in formal training (OR = 3.14, *P* < 0.05) were also positively associated with preventive practices.

**Discussion:**

These findings underscore the importance of structured assessment tools, ongoing education, and the need to foster positive attitudes among nurses to effectively reduce pressure ulcer prevalence and enhance patient outcomes in NICU and PICU settings.

## 1 Introduction

Pressure ulcers are a significant issue in neonatal and pediatric intensive care units (NICU & PICU), with their prevalence ranging from 0.8% to 27% globally. These ulcers pose substantial challenges for healthcare providers, contributing to increased morbidity, mortality, and healthcare costs. Pediatric patients at the highest risk include those requiring mechanical ventilation, inotropic support, or experiencing prolonged hospital stays and nutritional deficiencies. Medical device-related pressure ulcers are particularly common in this population, with prevalence rates between 50% and 69% (1, 2).

Pressure ulcers, also known as pressure sores or bedsores, are localized injuries to the skin and underlying tissue primarily caused by prolonged pressure or friction. These injuries typically occur over bony prominences such as the sacrum, heels, and hips. The National Pressure Ulcer Advisory Panel (NPUAP) and the European Pressure Ulcer Advisory Panel (EPUAP) categorize pressure ulcers into four stages based on severity, ranging from non-blanchable erythema of intact skin (Stage 1) to full-thickness tissue loss with exposed bone, tendon, or muscle (Stage 4). Factors contributing to the development of pressure ulcers include immobility, reduced skin sensitivity, and the use of medical devices (3).

Nursing strategies for pressure ulcer prevention encompass a range of evidence-based practices aimed at reducing the incidence and severity of these injuries. These strategies include regular repositioning of patients to alleviate pressure, the use of pressure-relieving devices such as specialized mattresses and cushions, meticulous skin care to maintain skin integrity, and thorough risk assessments using tools like the Braden Scale. Additionally, educational initiatives to enhance nurses’ knowledge and attitudes towards pressure ulcer prevention play a crucial role in implementing these strategies effectively. Implementing a multifaceted approach that combines these interventions has been shown to significantly reduce the incidence of pressure ulcers in both adult and pediatric populations (4, 5).

Globally, the prevalence of pressure ulcers in pediatric patients remains a critical concern, particularly in intensive care settings. A recent retrospective study conducted by Semerci et al. (6) analyzed data from 6,350 pediatric patients admitted to a university hospital between January 2019 and April 2022. The study found that the overall prevalence of pressure injuries (PIs) in the hospitalized pediatric population was 2.25%, with a significantly higher prevalence of 6.04% among patients in the Pediatric Intensive Care Unit (PICU). Notably, 21% of these patients had medical device-related pressure injuries (MDRPIs). The most common sites for PIs were the occiput (35.7%) and the coccyx/sacrum (13.3%), with 67.1% of the injuries classified as Deep Tissue Injuries. The study also identified several significant risk factors for PIs, including albumin level, hemoglobin level, Pediatric Nutrition Risk Score (PNRS), Body Mass Index (BMI), and length of hospital stay. These findings highlight the need for targeted preventive interventions, especially for MDRPIs, to improve patient outcomes in pediatric care settings (6).

A pressure ulcer (PU) or pressure sore is a localized injury to the skin and underlying tissue, primarily caused by prolonged pressure. Factors contributing to PUs include immobility, reduced skin sensitivity, and the use of medical devices. The National Pressure Ulcer Advisory Panel (NPUAP) and European Pressure Ulcer Advisory Panel (EPUAP) have identified these factors as key contributors (3). The prevalence of PUs varies widely, with rates of 27.0% for neonates, 19.2% for children under one year, and 12.3% for children older than one year (7, 8). PUs can range from superficial abrasions to severe injuries involving muscle and bone (9).

Pressure ulcers are categorized into four stages based on their severity. Stage 1 ulcers present as non-blanchable erythema of intact skin, while stage 2 ulcers involve partial-thickness loss of dermis. Stage 3 ulcers are characterized by full-thickness tissue loss, and stage 4 ulcers exhibit full-thickness tissue loss with exposed bone, tendon, or muscle (10, 11). Management of pressure ulcers includes debridement, infection control, moisture management, and use of appropriate dressings. Preventive measures involve regular repositioning, use of pressure-relieving devices, and maintaining skin hygiene (12, 13).

Preventing pressure ulcers is crucial for pediatric nurses. Studies indicate that nurses’ attitudes and behaviors significantly influence prevention practices (14). The quality of care provided by nurses is directly related to the development of pressure ulcers in patients (15, 16). Effective prevention in ICUs requires a multifaceted approach. Implementing evidence-based guidelines has proven to significantly reduce the incidence of pressure ulcers. For instance, a recent study demonstrated a 69% reduction in ICU-acquired pressure ulcers through a program involving risk assessment with the Braden scale, skin care protocols, and silicone gel adhesive dressings (17). Another study highlighted the effectiveness of regular repositioning and specialized mattresses in reducing pressure ulcer incidence (18).

Despite the availability of evidence-based guidelines, the practical implementation of pressure ulcer prevention strategies in NICU and PICU settings is often hindered by several barriers. These include insufficient staffing, which limits the ability to perform frequent patient repositioning, and time constraints that challenge thorough risk assessments and the consistent application of preventive measures. Moreover, resource limitations, such as the lack of adequate pressure-relieving devices, further impede effective prevention. Behavioral factors also play a significant role, as nurses’ perceptions of the importance of pressure ulcer prevention can vary, leading to inconsistent practices. For instance, in high-stress environments, prevention may be deprioritized in favor of more immediate patient care needs, particularly if nurses perceive pressure ulcer prevention as less critical. Additionally, the lack of ongoing training and education contributes to gaps in knowledge and skills, making it difficult for nursing staff to stay updated with the latest best practices. Addressing these barriers and fostering a proactive attitude toward pressure ulcer prevention are essential for improving patient outcomes in these critical care settings.

Research shows that pressure ulcers are as prevalent in the pediatric population as in adults, highlighting the importance of targeted preventive measures (19, 20). However, there is limited research focusing on the specific strategies used by nurses in NICU and PICU settings in Saudi Arabia. Previous studies in Saudi Arabia have highlighted high prevalence rates of pressure ulcers and the need for improved prevention strategies. This study aims to fill this gap by analyzing current nursing strategies, identifying barriers, assessing attitudes, and providing recommendations to improve nursing practices. By achieving these objectives, the study seeks to enhance the quality of care for pediatric patients and contribute to the broader knowledge of pressure ulcer prevention in critical care settings.

## 2 Materials and methods

### 2.1 Design

A descriptive study design with a cross-sectional survey method was employed in this study. This design was chosen because it is ideally suited for capturing and documenting the current state of nursing strategies for pressure ulcer prevention in neonatal and pediatric intensive care units. Descriptive studies allow for a detailed examination of existing practices, attitudes, and barriers within a specific population at a single point in time, providing an accurate snapshot of the current situation. The cross-sectional survey method enables efficient and cost-effective data collection from participants, which is particularly advantageous in clinical settings where time and resources are limited. Additionally, this design supports the identification of correlations and patterns that may inform practical recommendations for improving pressure ulcer prevention strategies.

### 2.2 Sample and sampling technique

A comprehensive sample of all nurses working in the neonatal and pediatric intensive care units (NICU & PICU) at King Fahad Medical City (KHMC), Riyadh, Saudi Arabia, was chosen, comprising a total of 80 nurses. The sample size for this study was determined based on the specific context and logistical constraints of conducting research in a high-demand clinical environment like the NICU and PICU. Given the specialized nature of care in these units, the number of available and eligible nursing staff was inherently limited. Additionally, the intense workload and critical responsibilities of nurses in these settings necessitated a manageable sample size to ensure the study's feasibility without compromising patient care. Despite the small sample size, the study aimed to provide in-depth insights and focused analysis.

### 2.3 Study size justification

The total number of nurses working in the NICU and PICU at King Fahad Medical City (KHMC), Riyadh, Saudi Arabia, was eighty. Given the specialized nature of care in these units, the number of available and eligible nursing staff was inherently limited. The intense workload and critical responsibilities of nurses in these settings necessitated a manageable sample size to ensure the study's feasibility without compromising patient care. Therefore, the entire population of eligible nurses was included to maximize the comprehensiveness and reliability of the findings. This approach aimed to provide in-depth insights and focused analysis that can be foundational for future research with larger and more diverse populations.

### 2.4 Inclusion criteria

Registered nurses working in the neonatal and pediatric intensive care units (NICU & PICU) at King Fahad Medical City (KHMC), Riyadh, Saudi Arabia, with more than one year of experience in these units, and who expressed interest in participating in the study, were included. The focus was on staff nurses who are directly involved in patient care. Head nurses, who primarily hold supervisory roles, were excluded from the study to ensure that the data reflects the practices and perspectives of those engaged in daily patient care activities.

### 2.5 Exclusion criteria

Nurses with less than one year of experience as a registered pediatric nurse in the NICU and PICU were excluded from the study.

### 2.6 Setting

The study was conducted in the Neonatal and Pediatric Intensive Care Units (NICU & PICU) at King Fahad Medical City (KHMC) in Riyadh, Kingdom of Saudi Arabia (KSA). KHMC is one of the largest and most advanced medical complexes in the Middle East, renowned for its high standards of care and specialized medical services. The hospital serves a large sector of the population in Riyadh, providing comprehensive healthcare to a diverse and extensive patient base. The choice of this setting is significant because KHMC serves as a leading healthcare institution in Saudi Arabia, providing cutting-edge treatments and comprehensive care to critically ill neonatal and pediatric patients. Furthermore, the insights gained from this study at KHMC are likely to be applicable to other similar settings, thus contributing to the broader knowledge of pressure ulcer prevention in NICU and PICU environments.

### 2.7 Tools of data collection

Two measurement instruments were employed for data collection. The nurse participants were responsible for filling out both the Nurses’ Profile Questionnaire, which gathered work-related data, and the Pressure Ulcer Survey Questionnaire.

(1) **The nurses’ profile questionnaire** included items related to the nurses’ work-related data, including their qualifications, years of experience, area of practice, presence of a pressure ulcer risk assessment tool in use in practice, presence of a pressure ulcer grading tool in use in practice, and formal training.
(2) **The pressure ulcer survey questionnaire** used in this study was adapted from Moore and Price's study in 2004 (21). Prior to its use, formal permission was requested and obtained from the original authors via email, following the procedures outlined for accessing and adapting content from limited access articles. The adaptation was conducted in accordance with the guidelines provided by the original authors.

#### 2.7.1 Section One: Pressure ulcer Prevention

It consisted of eleven items; four items were positive (one, six, seven, eleven), and seven items were negative (two, three, four, five, seven, eight, nine, ten, eleven). It was easy to administer, and the nurses could complete it successfully. Responses were collected using a five-point Likert-type scale, where five denoted “strongly agree”, four represented “agree”, three indicated “neither agree nor disagree”, two signified “disagree”, and one stood for “strongly disagree” for the positive items. For the negative items, the scores were reversed. To calculate the average score reflecting attitudes toward pressure ulcer prevention, responses from the Likert scales encompassing “strongly disagree”, “disagree”, and “neither agree nor disagree” were grouped as indicative of a negative attitude, while “strongly agree” and “agree” were considered indicative of a positive attitude. The nurses’ engagement in pressure ulcer prevention was observed to increase as the total mean scores rose. An overall attitude scores equal to or exceeding seventy percent was categorized as positive, while a total score below seventy percent was classified as negative, following the designated cutoff point value.

#### 2.7.2 Section Two: Pressure ulcer Behavior

In this section, the behavior of the nurses concerning pressure ulcer prevention was evaluated. The assessment tool comprised eight multiple-response items, encompassing inquiries about various aspects of pressure ulcer prevention, including pressure ulcer risk assessment (two items), the formulation of pressure ulcer prevention care plans (four items), and the implementation of pressure ulcer prevention strategies (two items).

#### 2.7.3 Section Three: Barriers Towards Pressure Ulcer Prevention

This section focused on evaluating the obstacles faced by nurses in the context of pressure ulcer prevention. The assessment tool comprised three multiple-response items, encompassing inquiries about specific challenges related to conducting pressure ulcer risk assessments (one item), documenting pressure ulcer prevention care plans (one item), and executing pressure ulcer prevention measures (one item).

### 2.8 Validity of the study tools

The content validity of the data collection tools was evaluated using a content validity index. A panel of three experts examined the inclusiveness and appropriateness of the items included to ascertain their clarity, comprehensibility, and relevance in achieving the study's objectives. The panel of three experts rated each item on a 4-point Likert scale, with 1 indicating “not relevant” and 4 indicating “highly relevant”. Based on this feedback, necessary adjustments were made to ensure the clarity and appropriateness of the tools. Adjustments were implemented as necessary to enhance the tools, and items with lower ratings were revised or removed to improve the validity of the instruments.

### 2.9 Reliability of study tools

Using Cronbach's α, the internal consistency reliability was found to be 0.71 for pressure ulcer prevention. The agreement percent for pressure ulcer behavior and barriers toward pressure ulcer prevention was 0.80 and 0.75, respectively.

### 2.10 Pilot study

A subset comprising ten percent of the subjects underwent testing with the data collection tools. These participants were subsequently excluded from the primary research study. The pilot study served the purpose of evaluating the suitability of the research instruments and estimating the time required to administer them. The insights gathered from the pilot study were instrumental in refining the tools, which involved corrections and additions to certain items. Following these adjustments, the final questionnaires were formulated.

A subset comprising ten percent of the subjects underwent testing with the data collection tools, and these participants were subsequently excluded from the primary research study. The pilot study served to evaluate the suitability of the research instruments and estimate the time required to administer them. Based on feedback from the pilot study, specific modifications were made to the questionnaires: “When do you carry out pressure ulcer risk assessment?” was revised to “For which patients do you carry out pressure ulcer risk assessments?” and also for the question “When do you carry out pressure ulcer risk assessment?” was clarified to “At what points during patient care do you carry out pressure ulcer risk assessments (e.g., upon admission, daily, or when a patient's condition changes)?” to ensure clarity. Additional response options were added to the question on barriers to implementing pressure ulcer prevention strategies, such as “lack of time” and “insufficient training”. The flow of questions was adjusted for better logical sequence and understanding, grouping questions on attitudes and following them with questions on the implementation of prevention strategies. These adjustments improved the clarity, comprehensiveness, and effectiveness of the data collection tools.

Several measures were taken to minimize potential biases in this study. Firstly, to reduce selection bias, a comprehensive sample of all eligible nurses working in the neonatal and pediatric intensive care units was included, ensuring a representative sample. Secondly, to mitigate information bias, we employed validated and reliable questionnaires that were translated and back-translated by two independent translators to ensure accuracy and consistency. Thirdly, to address potential response bias, we guaranteed anonymity and confidentiality to all participants, encouraging honest and uninfluenced responses. Additionally, a pilot study was conducted with 10% of the sample to identify and rectify any ambiguities in the questionnaires, further enhancing the reliability of the data collection tools.

These measures, along with the thorough validation and reliability testing, ensured that the data collection tools were robust and capable of providing reliable insights into nursing strategies for pressure ulcer prevention.

### 2.11 Data collection

All nurses working in the neonatal and pediatric intensive care units (NICU & PICU) at King Fahd Medical City (KHMC) in Riyadh, Saudi Arabia, were initially assessed for eligibility, totaling 80 nurses. Data were collected from these 80 nurses using the Nurses’ Profile Questionnaire and the Pressure Ulcer Survey Questionnaire, with all participants completing both questionnaires. Upon receiving the necessary permissions to proceed with the research proposal, the research team reached out to administrative nurses overseeing the paediatric and intensive care units to identify potential study participants. Subsequently, the nurses willingly agreed to take part in the study and were individually interviewed by the researchers to gain a comprehensive understanding of the study's objectives. The pressure ulcer survey questionnaire was administered to the nurses during their duty hours, and they were given 30–45 min to complete it. Data collection spanned from the start of February 2022 to the conclusion of March 2022, lasting over a period of two months. This timeline was chosen to accommodate the nurses’ work schedules and ensure a suitable timeframe for both the nurses and the respective units. Data from all 80 nurses were included in the analysis, with no dropouts or exclusions during the data collection phase, resulting in a final sample size of 80 nurses.

Several challenges were encountered during data collection, including coordinating with the nurses’ busy schedules and critical responsibilities, which made it difficult to find appropriate times for them to complete the questionnaires. To address this, data collection was scheduled during less busy hours and extended over two months to provide flexibility. Making sure the nurses fully understood the study's objectives was another challenge, addressed by conducting individual interviews to explain the study and answer questions. Additionally, potential response bias was reduced by emphasizing anonymity and confidentiality, encouraging honest and uninfluenced feedback. Despite these challenges, data from all 80 nurses were successfully collected and included in the analysis, with no dropouts or exclusions, resulting in a final sample size of 80 nurses.

### 2.12 Data analysis

Data analysis in this study was conducted using the Statistical Package for the Social Sciences (SPSS), Version 23 for Windows. Descriptive statistics were used to summarize the data, including means, standard deviations (SD), frequencies, and percentages. Inferential statistics, such as the Monte Carlo chi-squared test, were used to assess potential relationships between the demographic characteristics of the nurses and their implementation of pressure ulcer prevention measures. The Monte Carlo chi-squared test was chosen because it provides a more accurate *P*-value for small sample sizes or when the data distribution does not meet the assumptions of the traditional chi-squared test, making the results more reliable.

Additionally, Logistic Regression Analysis was performed to examine the relationships between various independent variables (e.g., nurse qualifications, employment duration, area of practice, presence of pressure ulcer risk assessment and grading tools, formal training, and attitudes towards pressure ulcer prevention) and the dependent variable (pressure ulcer prevention practices). For all statistical tests, a *P*-value of less than 0.05 was considered statistically significant. Using logistic regression analysis helped identify significant predictors of pressure ulcer prevention practices among nurses, while accounting for potential confounders. This method gives a more complete understanding of the factors influencing pressure ulcer prevention practices and helps identify key areas for targeted interventions. The results of the logistic regression analysis are presented in a table, showing the odds ratios (OR) and 95% confidence intervals (CI) for each predictor variable, thus offering deeper insights into the factors affecting effective pressure ulcer prevention in neonatal and pediatric intensive care settings.

Missing data were handled using appropriate statistical techniques to ensure the analysis was robust. In cases where data were missing completely at random (MCAR), the cases with missing values were excluded from the specific analyses involving those variables. For data not missing completely at random, multiple imputation methods were used to estimate and replace the missing values. This method allows for the retention of all cases by replacing missing values with a set of plausible values based on the observed data, thus preserving the sample size and reducing potential bias.

### 2.13 Ethical considerations

After obtaining permission from the Ethics Committee (permission code (2021/0026/IRB-21/6/2021), after acquiring the necessary permits and providing a letter of introduction, formal authorization for data collection was secured from the hospital's administrative staff. Written consent was obtained from the participating nurses, with the researchers providing a clear explanation of the study's purpose and objectives. Participants were granted the option to withdraw from the study at any point. To safeguard the nurses’ confidentiality and anonymity, measures were put in place.

## 3 Results

Table 1 shows that in terms of qualifications, 32.5% of the nurses had been qualified for more than ten years, 30% for two to less than six years, and 30% for six to less than ten years. 41.3% had been employed as a permanent staff nurse in the hospital for two to less than six years, followed by 27.3% for six to less than ten years. As for the area of practice, 58.5% had been working in the Neonatal Intensive Care Unit and 41.3% in the Paediatric Intensive Care unit. Moreover, 86.3% agreed to the presence of a pressure ulcer risk assessment tool in practice, and 72.5% agreed to the presence of a pressure ulcer grading tool in practice.

**Table 1:** Percentage distribution of the studied nurses according to their work-related data (*n* = 80).

Items	Total sample (*n* = 80)
How long have you been qualified as a nurse?	
Less than 2 years	6 (7.5%)
2-<6 years	24 (30.0%)
6-<10 years	24 (30.0%)
More than 10 years	26 (32.5%)
How long have you been employed as a permanent Staff Nurse in your hospital?	
Less than 2 years	10 (12.5%)
2-<6 years	33 (41.3%)
6-<10 years	22 (27.5%)
More than 10 years	15 (18.8%)
Mean years of experience	6.23 years
What area of practice do you work in?	
Neonatal Intensive Care Unit (NICU)	47 (58.8%)
PaediatricIntensive Care Unit (PICU)	33 (41.3%)
Is there a pressure ulcer risk assessment tool in use in your practice?	
Yes	69 (86.3%)
No	11 (13.8%)
Is there a pressure ulcer grading tool in use in your practice?	
Yes	58 (72.5%)
No	22 (27.5%)

**Figure 1:**
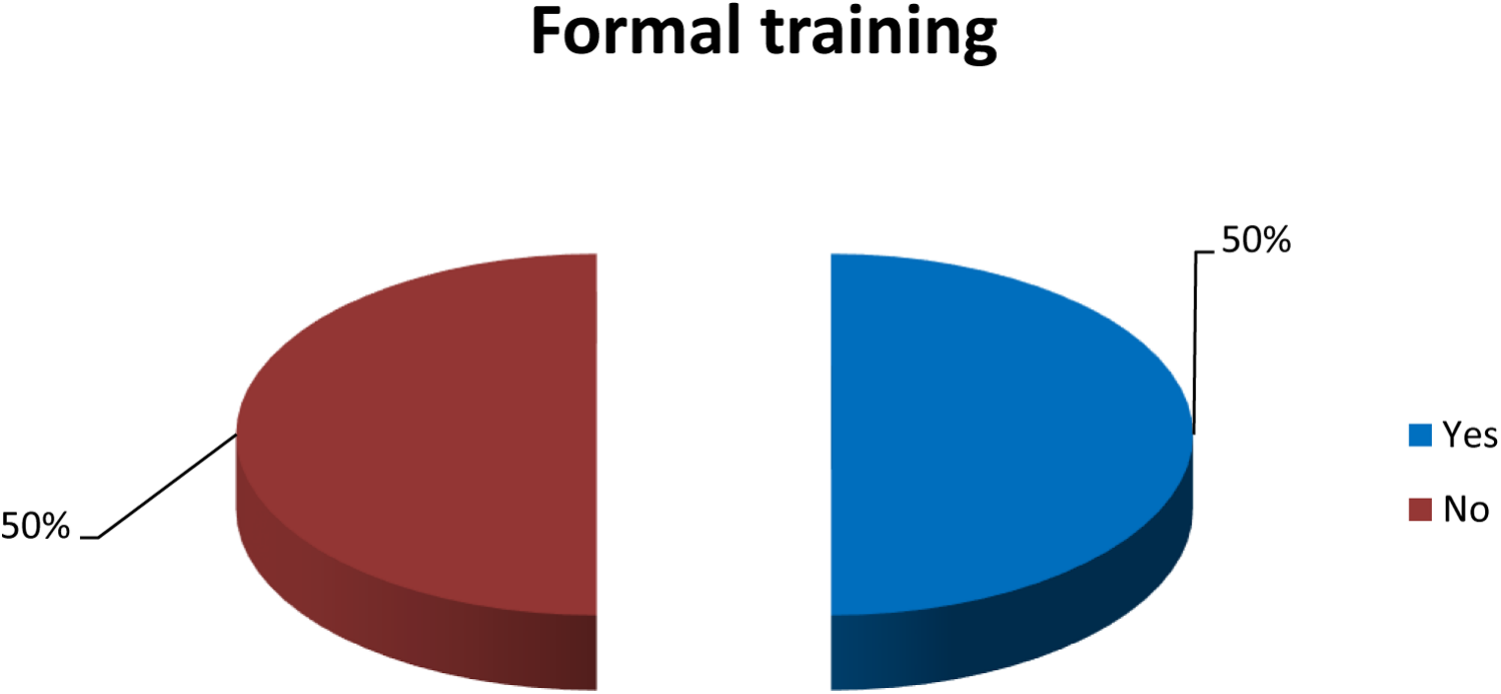
Distribution of studied nurses regarding formal training on pressure ulcer prevention & management (*n* = 80).

Figure 1 illustrates that 50% of the nurses in the study had received formal training on pressure ulcer prevention and management.

Table 2 reveals that the total attitude mean score was 3.57. The total mean score for the positive attitude items was 3.85, and the item “most pressure ulcers can be avoided item” garnered a mean score of 4.29. On the other hand, the total mean score for the negative items was 3.29. The item “pressure ulcer prevention is a low priority for me” had the highest mean score of 3.86, followed by “I do not need to concern myself with pressure ulcer prevention in my practice” (mean score = 3.78), “I am less interested in pressure ulcer prevention than other aspects of nursing care” (mean score = 3.61), and “pressure ulcer treatment is a greater priority than pressure ulcer prevention” (mean score = 3.55).

**Table 2:** Distribution of the studied nurses’ attitudes toward pressure ulcer prevention (*n* = 80).

Items	Mean ± SD
Positive items scores	
All inpatients are at potential risk of developing pressure ulcers	3.37 ± 1.05
Continuous nursing assessment of patients will give an accurate picture of their pressure ulcer risk	4.10 ± 0.62
Most pressure ulcers can be avoided.	4.29 ± 0.49
During a patient's hospital stay, it is essential to conduct routine pressure ulcer risk assessments for all patients	3.63 ± 1.72
Total mean score	3.85 ± 0.40
Negative items scores	
Pressure ulcer prevention is time-consuming for me to carry out	2.59 ± 1.04
In my opinion, patients tend not to get as many pressure ulcers nowadays	2.65 ± 0.95
I do not need to concern myself with pressure ulcer prevention in my practice	3.78 ± 0.92
Pressure ulcer treatment is a greater priority than pressure ulcer prevention	3.55 ± 1.14
I am less interested in pressure ulcer prevention than in other aspects of nursing care	3.61 ± 0.84
My clinical judgment is better than any pressure ulcer risk assessment tool available to me	2.96 ± 0.98
Compared to other aspects of nursing care, I consider pressure ulcer prevention to be a lower priority.	3.86 ± 0.91
Total mean score	3.29 ± 0.95
Total attitude mean score	3.57 ± 0.24

**Figure 2:**
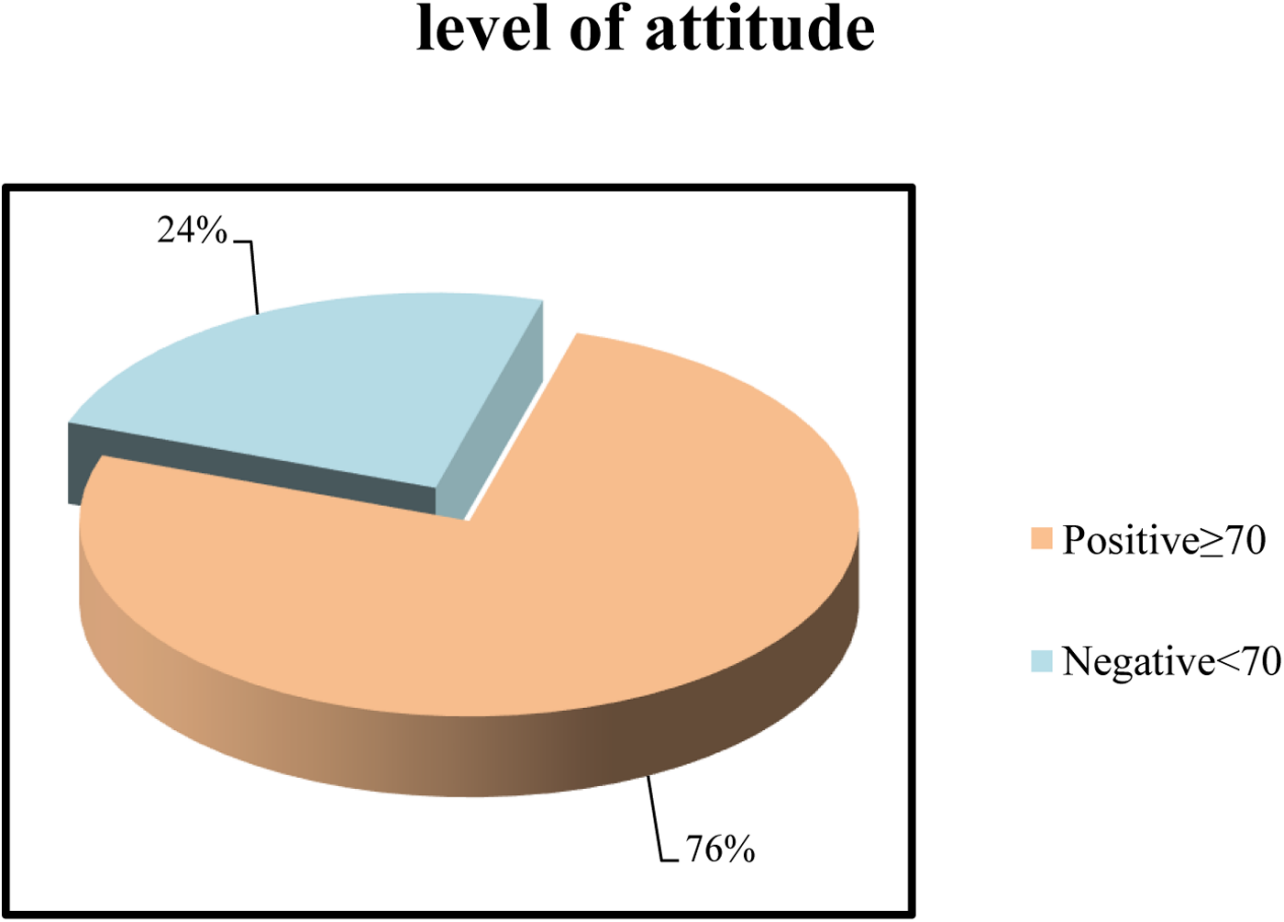
Level of attitude of the studied sample (*n* = 80).

Figure 2 illustrates that 76% of studied nurses had a positive attitude toward pressure ulcer prevention, while 24% had a negative attitude toward pressure ulcer prevention.

Table 3 shows that 72.5% claimed to carry out pressure ulcer risk assessment on all patients. 67.5% of the studied nurses had completed a pressure ulcer risk assessment daily, and 60% had written a pressure ulcer prevention care plan. Furthermore, 46.3% had read the patients’ pressure ulcer prevention care plans daily, and 47.3% had a cause for reviewing the care plan, followed by 30% who saw the changes in the patient's condition. Moreover, 57.5% of the participants had updated the patient pressure ulcer prevention care plans daily during the patient's stay in the hospital, and 36.3% had updated the plan only when the patients developed a pressure ulcer during their stay in the hospital. Lastly, 76.3% of the sample had carried out pressure ulcer prevention strategies, of which 87.5% did so because they felt they were an essential part of nursing practice.

**Table 3:** Percentage distribution of nurses about pressure ulcer behavior (*n* = 80).

Items	*N*	%
For which patients do you carry out pressure ulcer risk assessments		
On all patients	58	72.5
On some patients	19	23.8
On no patients	3	3.8
At what points during patient care do you carry out pressure ulcer risk assessments		
On admission only	6	7.5
Daily during the patient's stay in the hospital	54	67.5
Only when the patient develops a pressure ulcer during their stay in the hospital	14	17.5
When I remember to	6	7.5
When I get time	0	0
When do you write a pressure ulcer prevention care plan?		
On all patients at risk	48	60.0
On some patients at risk	30	37.5
On no patients	2	2.5
When do you read patients’ pressure ulcer prevention care plans?		
Daily	37	46.3
Weekly	23	28.8
Less often	16	20.0
Never	4	5.0
Why do you read patients’ pressure ulcer prevention care plans?		
To review the care plan	38	47.5
Because there is a change in the patient's condition	24	30.0
Because the patient has developed a pressure ulcer	16	20.0
Others	2	2.5
When do you update patients’ pressure ulcer prevention care plans?		
Daily during the patient's stay in hospital	46	57.5
Only when the patient develops a pressure ulcer during his/her stay in hospital	29	36.3
When I remember to	0	0
Never	5	6.3
Other—please specify:	0	0
Do you ever carry out pressure ulcer preventative strategies?		
Yes	61	76.3
No	19	23.8
Why do you carry out pressure ulcer preventative strategies?		
Because they are an essential part of nursing practice	70	87.5
Because I see other nurses doing the same	2	2.5
Because other nurses expect me to	3	3.8
Because the hospital policy states that I should	3	3.8
Others	2	2.5

**Figure 3:**
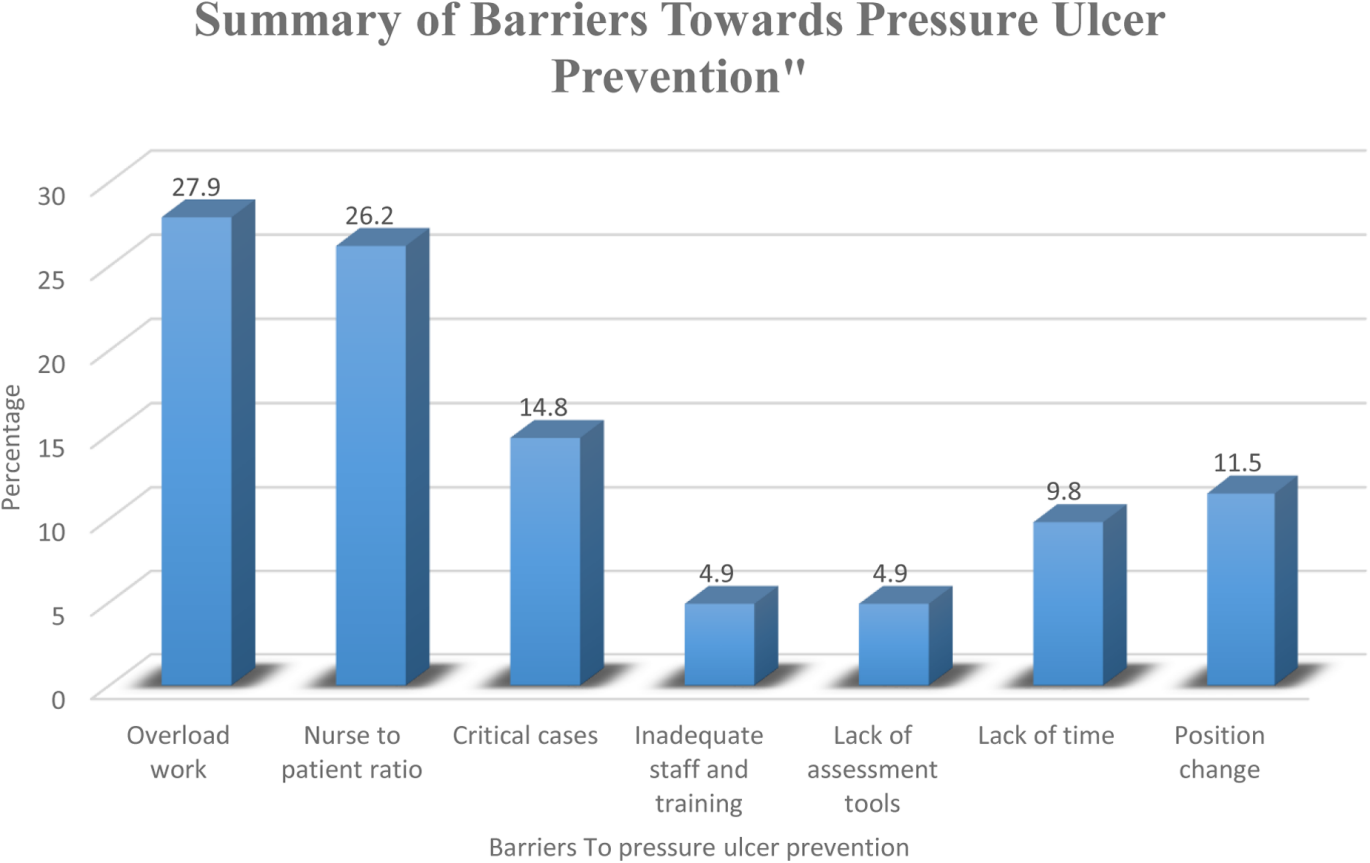
Summary of barriers towards pressure ulcer prevention (*n* = 80).

Figure 3 illustrates the various barriers identified by nurses in preventing pressure ulcers in neonatal and pediatric intensive care units (NICU & PICU). The most prevalent barrier to pressure ulcer prevention reported by the nurses was “Overload work”, identified by 27.9% of the respondents as a significant challenge. This was closely followed by “Nurse to patient ratio”, cited by 26.2% of the nurses. “Critical cases” were also a notable barrier, mentioned by 14.8% of the nurses, indicating the high demands of caring for severely ill patients. Conversely, the least reported barriers were “Inadequate staff and training” and “Lack of assessment tools”, each reported by 4.9% of the nurses. Additionally, 9.8% of the nurses identified “Lack of time” as a barrier, while “Position change” was reported by 11.5% of the nurses.

Table 4 reveals that 76.2% of the respondents agreed that there were barriers toward carrying out pressure ulcer risk assessment. 27.9% indicated it was overwork, and 26.2% considered the nurse-to-patient ratio a leading cause. Also, barriers to documenting the pressure ulcer prevention care planning were also highlighted by 67.5%. 54.6% considered heavy workload to be the main cause, followed by the pressure of time, as indicated by 14.5% of the nurses. Moreover, 65% thought there were barriers to carrying out a pressure ulcer prevention plan. 65.5% thought that the main cause was heavy workload, followed by inadequate staff (11.5%) and pressure of time (9.6%).

**Table 4:** Percentage distribution of barriers towards pressure ulcer prevention (*n* = 80).

Items	*N*	%
Carrying out pressure ulcer risk assessment		
Yes	61	76.2
No	19	23.8
If yes (*n* = 61)		
Overload work	17	27.9
Nurse to patient ratio	16	26.2
Critical cases	9	14.8
Inadequate staff and inadequate training	3	4.9
Lack of assessment tools	3	4.9
Lack of time	6	9.8
Position change	7	11.5
Documenting pressure ulcer prevention care planning		
Yes	55	67.5
No	25	37.5
If yes (*n* = 55)		
Heavy workload	30	54.6
Unstable and serious condition	2	3.6
Inadequate nutrition	2	3.6
Daily assessment	6	10.9
Pressure of time	8	14.5
Inadequate training	2	3.6
Agitated and mobile patient	3	5.6
Poor documentation	2	3.6
Do you carry out a pressure ulcer prevention plan?		
Yes	52	65
No	28	35
If yes (*n* = 52)		
Heavy workload	34	65.5
Inadequate staff	6	11.5
Lack of training	2	3.8
Work stress	3	5.8
Lack of supplies	2	3.8
Pressure of time	5	9.6

Table 5, shows that there was a statistically significant difference between carrying out pressure ulcer prevention and nurse qualifications with P.005* and carrying out pressure ulcer prevention and employment as a permanent staff nurse with P.006*.

**Table 5:** Relation between demographic characteristics and carrying out pressure ulcer prevention.

Items	Carrying out pressure ulcer prevention	*X*^2^ mc (*P* value)
How long have you been qualified as a nurse?		
Less than 2 years	Yes: 6 (100%) No: 0 (0%)	13.02 (.005*)
2-<6 years	Yes: 16 (66.7%) No: 8 (33.3%)	
6-<10 years	Yes: 14 (58.3%) No: 10 (41.7%)	
More than 10 years	Yes: 25 (96.2%) No: 1 (3.8%)	
How long have you been employed as a permanent Staff Nurse in your hospital?		
Less than 2 years	Yes: 10 (100%) No: 0 (0%)	11.47 (.006*)
2-<6 years	Yes: 21 (63.6%) No: 12 (36.4%)	
6-<10 years	Yes: 15 (68.2%) No: 7 (31.8%)	
More than 10 years	Yes: 15 (100%) No: 0 (0%)	

MC is Monte Carlo for Chi square test and Significant at *P* < 0.05.

* Denotes statistical significance at the *P* < 0.05 level.

Table 6, presents the results of a multivariate analysis examining factors influencing pressure ulcer prevention practices among nurses. The logistic regression analysis highlights several key predictors. Nurses with more than 10 years of qualification have significantly higher odds (OR = 3.67) of engaging in pressure ulcer prevention compared to those with less than 2 years of qualification, though the wide confidence interval suggests variability. Similarly, permanent staff nurses with over 10 years of employment also show increased odds (OR = 4.31) for pressure ulcer prevention. The presence of a pressure ulcer grading tool (OR = 2.49, *P* < 0.05) and formal training in pressure ulcer prevention (OR = 3.14, *P* < 0.05) are both significant factors, indicating that structured assessment tools and education significantly improve prevention practices. Nurses with positive attitudes towards pressure ulcer prevention are also more likely (OR = 2.78, *P* < 0.05) to engage in preventive measures.

**Table 6:** Multivariate analysis of factors influencing pressure ulcer prevention practices.

Variable	Categories	Pressure ulcer prevention practices (%)	Logistic regression analysis (odds ratio [OR], 95% confidence interval [CI])
How long have you been qualified as a nurse?	Less than 2 years	100%	Reference Group
	2-<6 years	66.7%	0.58 (0.12–2.79)
	6-<10 years	58.3%	0.48 (0.10–2.34)
	More than 10 years	96.2%	3.67 (0.41–32.97)
How long have you been employed as a permanent Staff Nurse in your hospital?	Less than 2 years	100%	Reference Group
	2-<6 years	63.6%	0.54 (0.12–2.43)
	6-<10 years	68.2%	0.64 (0.13–3.05)
	More than 10 years	100%	4.31 (0.46–40.57)
What area of practice do you work in?	NICU	72.3%	1.28 (0.50–3.31)
	PICU	81.8%	Reference Group
Is there a pressure ulcer risk assessment tool in use in your practice?	Yes	78.3%	2.12 (0.58–7.70)
	No	54.5%	Reference Group
Is there a pressure ulcer grading tool in use in your practice?	Yes	82.8%	2.49 (1.28–4.83)*
	No	50.0%	Reference Group
Formal training on pressure ulcer prevention & management	Yes	84.0%	3.14 (1.67–5.91)*
	No	45.8%	Reference Group
Attitudes towards pressure ulcer prevention (mean score)	Positive (≥70%)	89.1%	2.78 (1.50–5.15)*
	Negative (<70%)	40.9%	Reference Group

* Indicates statistically significant predictors (*P* < 0.05).

## 4 Discussion

Pressure ulcer prevention is considered one of the most significant quality indicators of patient care in healthcare. Timely nursing interventions have a crucial effect on pressure ulcer avoidance and development, representing an important nurse-sensitive issue (22). This study aimed to understand the nurses’ attitudes, behaviors, and perceived barriers to PU prevention in patients. Positive attitudes regarding pressure ulcer prevention are crucial for providing excellent nursing care. They enable nurses to use sufficient and appropriate preventive actions for high-risk persons, ensuring a decrease in the incidence rates of PU, hospital stays, morbidity and mortality rates, and the cost of care (23).

The findings of the study indicated a notably high mean score for attitudes. Among the positive attitude items, the item “most pressure ulcers can be avoided” had the highest mean score. The high mean score for attitudes could be attributed to the implementation of national pressure ulcer prevention guidelines in hospitals, which likely raised awareness among nurses about the importance of preventive measures. Additionally, this could reflect the effectiveness of continuous training programs that emphasize pressure ulcer prevention as a core nursing competency. Regarding negative attitude items, the study identified that the statement “pressure ulcer prevention is a low priority for me” had the highest mean score, followed by “I do not need to concern myself with pressure ulcer prevention in my practice”, “I am less interested in pressure ulcer prevention than other aspects of nursing care”, and “pressure ulcer treatment is a greater priority than pressure ulcer prevention”. These results were consistent with other studies that have reported a high total attitude mean score (22, 24). The presence of negative attitudes among some nurses may indicate a perception of pressure ulcer prevention as time-consuming or secondary to more immediate clinical tasks. This perception could be influenced by factors such as workload, inadequate staffing, and competing priorities within the healthcare environment, where nurses often need to manage multiple urgent tasks simultaneously.

In the same context, the research findings indicated that a significant majority of the nurses under study exhibited a positive attitude, aligning with the conclusions of prior studies that also reported a positive overall attitude (25–27). These findings could be interpreted in light of the fact that nurses inherently have positive attitudes toward performing these actions. Conversely, these findings contradict other studies that have reported a negative attitude among nurses (28, 29).

Concerning the nurses’ behaviors toward pressure ulcer prevention, the study results showed that most nurses carried out pressure ulcer risk assessments on all patients daily, and two-thirds had written a pressure ulcer prevention care plan. These proactive behaviors can likely be linked to institutional protocols mandating routine assessments for pressure ulcer risk, especially in high-risk units like NICUs and PICUs. Furthermore, the regular review and update of prevention care plans may reflect a high level of accountability and compliance with hospital policy, where nurses are expected to take a preventative approach to patient care. Additionally, less than half had read patients’ pressure ulcer prevention care plans daily, 47.3% had reviewed them when there was a cause, and 30% did so after observing a change in the patient's condition. Furthermore, more than half of the studied nurses had updated patients’ pressure ulcer prevention care plans daily during the patient's stay in the hospital, and the majority of the sample had carried out pressure ulcer preventive strategies, acknowledging their essential role in nursing practice.

These findings could be explained by the fact that individual action is affected by the person's attitude toward certain behaviors, and PU prevention behaviors had the highest priority among the entire sample. This interpretation is supported by the Theory of Planned Behavior, which posits that behaviors stem from beliefs that a particular behavior contributes to a particular effect (30). The alignment with the Theory of Planned Behavior suggests that nurses’ attitudes toward pressure ulcer prevention are not only influenced by their personal beliefs but also by institutional and social norms that value preventive care. The high priority placed on pressure ulcer prevention in critical care settings likely reinforces these behaviors, as nurses are regularly reminded of the consequences of neglecting these preventive measures.

Regarding barriers to pressure ulcer prevention, the study results showed that most of the nurses highlighted the existence of barriers to conducting a pressure ulcer risk assessment, with workload and nurse-to-patient ratios being the main hindrances. More than two-thirds of nurses noted barriers to documenting pressure ulcer prevention care plans, with more than half attributing these barriers to a heavy workload, inadequate staff, and time pressure. These results align with global findings that staff shortages hinder hospital staff from practicing assessment techniques, decreasing the time available to practice patient health care and deliver high-quality care relevant to pressure ulcer prevention (16, 31–33). Workload and nurse-to-patient ratios are particularly relevant barriers in critical care settings, where patient acuity is high, and the demands on nursing staff are intense. In Saudi healthcare facilities, these barriers may be exacerbated by staffing shortages and the rapid turnover of patients, requiring nurses to prioritize tasks based on immediate patient needs rather than preventive actions.

The study also found a statistically significant difference between carrying out pressure ulcer prevention and nurse qualifications, as well as employment as a permanent staff nurse. These findings are consistent with previous study results, which reported that nurses’ experience significantly affected their attitudes regarding pressure ulcer prevention (34, 35).

Comparing these findings to the study by Amr et al., there are similarities and differences. The study titled “A Pre-Post Study Evaluating the Effectiveness of a New Initiative, the “PRESSURE Bundle,” Compared with Standard Care in Reducing the Incidence and Prevalence of Sacral Pressure Ulcers in Critically Ill Patients in an Intensive Care Unit in Riyadh, Saudi Arabia” also emphasised the importance of systematic preventive measures. The “PRESSURE Bundle” significantly reduced the incidence and prevalence of sacral pressure ulcers through structured interventions, including positioning, risk assessment, and skin care protocols (36). This reinforces the necessity of comprehensive, evidence-based strategies in pressure ulcer prevention, highlighting the role of structured protocols and continuous monitoring in achieving significant improvements in patient outcomes.

The results of this study underscore the importance of structured tools and formal training in enhancing pressure ulcer prevention practices. Specifically, the significant odds ratio for the presence of a pressure ulcer grading tool (OR = 2.49, 95% CI = 1.28–4.83) indicates that nurses with access to such tools are better equipped to prevent pressure ulcers. This finding is in line with previous research by Zhang et al. (37), which demonstrated that the implementation of standardized risk assessment tools significantly reduced the incidence of pressure ulcers in hospital settings (37).Additionally, the study's finding that formal training on pressure ulcer prevention significantly improves prevention practices (OR = 3.14, 95% CI = 1.67–5.91) aligns with the results of a study by Stansby et al. (38), which emphasized the critical role of continuing education in maintaining high standards of clinical practice and improving patient outcomes (38).

Moreover, the positive correlation between nurses’ attitudes towards pressure ulcer prevention and their prevention practices (OR = 2.78, 95% CI = 1.50–5.15) highlights the importance of fostering a positive work culture. This finding is consistent with the results of a study by Samuriwo and Dowding (39), which found that nurses with positive attitudes towards pressure ulcer prevention were more likely to engage in proactive preventive measures (39). However, despite these positive correlations, the study also revealed significant barriers such as heavy workload and inadequate staffing, which are consistent with findings from a global survey by Moore and Patton (40) that identified similar challenges across various healthcare settings. These barriers suggest that while training and tools are essential, organizational support and adequate staffing are also crucial for effective pressure ulcer prevention (40).

Most of the studied nurses had a positive attitude toward pressure ulcer prevention, with less than one-third exhibiting a negative attitude. The majority of nurses carried out pressure sore risk assessments on all of their patients and identified staff shortages and time constraints as the main barriers to conducting these assessments. Multivariate analysis revealed several key predictors of pressure ulcer prevention practices. Nurses with more than 10 years of qualification had significantly higher odds of engaging in pressure ulcer prevention. Permanent staff nurses with over 10 years of employment also showed increased odds for pressure ulcer prevention. The use of a pressure ulcer grading tool and formal training in pressure ulcer prevention were significant factors that improved prevention practices. Additionally, nurses with positive attitudes towards pressure ulcer prevention were more likely to engage in preventive measures.

To address the barriers identified, it is recommended to provide in-service training for nurses on pressure ulcer prevention, supported by evidence-based guidelines. The implementation of structured assessment tools, such as pressure ulcer grading tools, and formal training programs significantly enhance prevention practices. This study contributes to the body of research by providing a comprehensive analysis of nursing strategies for pressure ulcer prevention in neonatal and paediatric intensive care units in Saudi Arabia. It highlights the critical role of nurses’ attitudes and behaviors in implementing preventive measures and identifies specific barriers that hinder effective prevention. The findings underscore the need for targeted training and resource allocation to improve pressure ulcer prevention practices in these settings.

One of the key strengths of this study is its focus on pressure ulcer prevention in specialized care environments such as NICU and PICU. These units present unique challenges due to the vulnerable nature of the patient population, and this study directly addresses the specific strategies used by nurses in these settings. Additionally, the use of validated tools for assessing nurse attitudes, behaviors, and barriers to pressure ulcer prevention ensures that the findings are both reliable and robust. The study also provides valuable multivariate analysis, identifying significant predictors of effective pressure ulcer prevention, such as the number of years’ nurses have been qualified, the availability of structured assessment tools, and formal training programs. These factors emphasize the importance of experience and education in enhancing nursing practices. Furthermore, the study highlights organizational factors, like staffing and workload, that can impact the successful implementation of prevention strategies. This in-depth exploration of both individual and institutional influences on pressure ulcer prevention contributes to a comprehensive understanding of how to improve patient care in critical care environments.

Future research should explore the long-term effects of enhanced training programme on pressure ulcer prevention outcomes in neonatal and paediatric intensive care units. Studies should also investigate the impact of organizational changes, such as improved nurse-to-patient ratios and the implementation of advanced technological solutions, on the prevalence of pressure ulcers. Additionally, research should be conducted in diverse healthcare settings to generalize the findings and develop a comprehensive framework for pressure ulcer prevention across different populations.

## 5 Limitations

This study has some limitations. The sample size was relatively small and confined to a single medical center, which may limit the generalisability of the findings. The sample size of this study was determined based on the specific context and logistical constraints of conducting research in a high-demand clinical environment like the Neonatal and Paediatric Intensive Care Units. Given the specialized nature of care in these units, the number of available and eligible nursing staff was inherently limited. Furthermore, the intense workload and critical responsibilities of nurses in these settings necessitated a manageable sample size to ensure the study's feasibility without compromising patient care. Additionally, the study relied on self-reported data, which may be subject to response bias. Future studies should aim to include larger and more diverse samples, as well as objective measures of pressure ulcer prevention practices and outcomes. Despite these limitations, the study provides in-depth insights and a focused analysis, serving as a foundation for future research.

## Data Availability

The datasets generated and analyzed during the current study are available from the corresponding author upon reasonable request.
